# MiR-320a inhibits gastric carcinoma by targeting activity in the FoxM1-P27^KIP1^ axis

**DOI:** 10.18632/oncotarget.8676

**Published:** 2016-04-11

**Authors:** Yangyang Wang, Jiping Zeng, Jianyong Pan, Xue Geng, Lupeng Li, Jing Wu, Ping Song, Ying Wang, Jilan Liu, Lixiang Wang

**Affiliations:** ^1^ Department of Pharmacology, Shandong University School of Medicine, Jinan 250012, P.R.China; ^2^ Department of Biochemistry and Molecular Biology, Shandong University School of Medicine, Jinan 250012, P.R. China; ^3^ Department of Hepatobiliary Surgery, Qilu Hospital of Shandong University, Jinan 250012, P.R. China

**Keywords:** miR-320a, FoxM1, proliferation, gastric cancer, P27^KIP1^

## Abstract

MicroRNAs (miRNAs) regulate tumorigenesis by inhibiting gene expression. In this study, we showed that miR-320a expression is decreased in human gastric cancer tissues and correlates inversely with expression of FoxM1, a key cell cycle regulator involved in gastric carcinoma. By contrast, the expression of P27^KIP1^, a downstream effector of FoxM1, correlates positively with miR-320a levels. Luciferase assays indicate that miR-320a suppresses FoxM1 expression, and *in vitro* recovery tests using FoxM1 siRNA indicate miR-320a inhibits gastric cancer cell proliferation by suppressing activity in the FoxM1-P27^KIP1^ axis. *In vivo*, nude mice injected with BGC-823 gastric cancer cells expressing a miR-320a inhibitor exhibit faster tumor growth than mice injected with control cells. Analysis of FoxM1 and P27^KIP1^ expression in tumor tissues indicates that miR-320a suppression increases the tumor growth by enhancing FoxM1-P27^KIP1^ signaling. These results thus reveal the crucial role played by miR-320a in limiting gastric carcinoma by directly targeting FoxM1- P27^KIP1^ axis.

## INTRODUCTION

Gastric cancer is the fourth most commonly diagnosed cancer and the second leading cause of cancer death worldwide, especially in developing countries [1, 2]. Although the incidence has gradually decreased, there are about 700,000 confirmed mortalities annually worldwide [3]. Gastric cancer is generally diagnosed at an advanced stage, which is the primary cause of its poor prognosis [4]. Infection with *Helicobacter pylori* is one of the most important factors contributing to the development of gastric carcinoma [5]. In order to improve the outcome of gastric cancer, identification of genetic and epigenetic events regulating the proliferation of gastric cancer cells is required. System biological analyses of gastric cancer samples have shown the importance of microRNAs (miRNAs) in this process [6, 7].

MiRNAs are small noncoding RNAs with 18-24 nucleotides in length [8]. They have emerged as important posttranscriptional regulators, which negatively regulate gene expression by directly targeting the three prime untranslated region (3′-UTR) of mRNAs, either promoting the degradation of target mRNAs or preventing their translation [9, 10]. MiRNAs are estimated to regulate up to 30% of genes in the human genome [11]. Over the past few years, studies of miRNAs have revealed that miRNAs participate in regulation of various biological processes, such as cell proliferation, apoptosis, and differentiation, as well as tumor development, metastasis, angiogenesis, and immune responses. MiRNAs play important roles in tumorigenesis [12, 13]. Some miRNAs act as tumor suppressors or oncogenes, depending on the function of their target genes [14, 15]. Recent studies also indicate that some miRNAs contribute to gastric carcinoma, including activated miRNAs (such as miR-21, miR-107, miR-222, and miR-106b) and suppressed miRNAs (such as miR-143, miR145, miR-622, and miR-148a) [16]. We have previously found that gastric carcinoma samples have activated several signal pathways, including the Forkhead box M1 (FoxM1) signaling pathway, which includes special miR-320a [17, 18]. MiR-320a has been identified to act as a tumor suppressor in some types of cancers, such as colon cancer, breast cancer, and bladder cancer [19–21]. More work needs be done to elucidate the role of miR-320a in gastric carcinoma.

FoxM1 is a member of the Fox transcription factor family, which is a key stimulator of cell proliferation and functions as an oncogene [22, 23]. We have previously reported that FoxM1 is activated in gastric cancer and that it induces gastric cancer cell proliferation by the inhibition of P27^KIP1^ [17]. P27^KIP1^ is an important negative molecular checkpoint [24]. In this study, we aimed to identify the role of miR-320a in gastric carcinoma and the down-stream FoxM1 and P27^KIP1^ regulatory mechanisms both *in vitro* and *in vivo*.

## RESULTS

### Human gastric cancer tissues exhibit low miR-320a expression and increased FoxM1 expression

**Figure 1:**
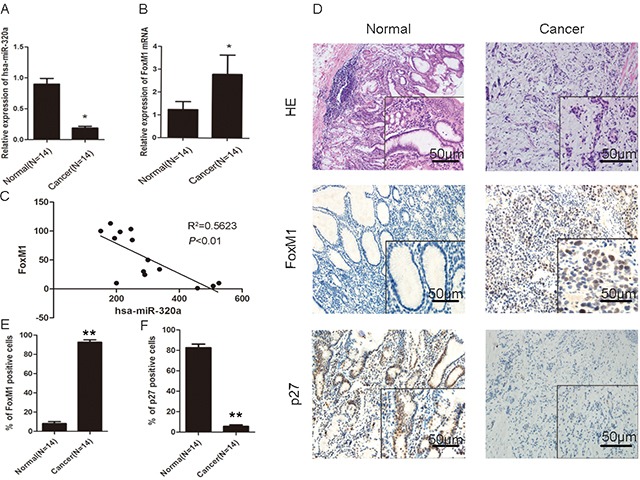
Association of miR-320a reduced expression and increased FoxM1 expression with the inhibition of P27^KIP1^ in human gastric cancer QRT-PCR analyses of **A.** miR-320a and **B.** FoxM1 mRNA in normal and cancerous human gastric tissues. *P < 0.05. **C.** Correlation of miR-320a and FoxM1levels in human gastric cancer tissues after standardization with matched normal tissues. **P < 0.01. **D.** HE staining and IHC staining of FoxM1 and P27^KIP1^ in human normal (left panel) and cancerous (right panel) gastric tissues. Representative images are shown. **E.** Percentage of FoxM1 and P27^KIP1^ positive cells analyzed by IHC in human normal and cancerous gastric tissues. **P < 0.01. Data are mean ± SEM of 3 independent experiments.

We carried out microdissection to obtain gastric epithelial cells from gastric cancer and normal tissues, and extracted RNA for microarray analysis. By analyzing the microarray data, we found that some important signaling pathways, such as NF-κB, Wnt/β-catenin, Ras-MAPK, and FoxM1 were activated in gastric carcinoma. Bioinformatics databases [miRanda (http://www.microrna.org), TargetScan (http://www.targetscan.org), and miRBase (http://www.mirbase.org)] predicted that miR-320a is an up-stream regulator of FoxM1. Compared with normal human tissues, gastric cancer specimens showed significant inhibition of miR-320a expression and activation of FoxM1 (Figure 1A, 1B and 1C). By qRT-PCR and immunohistochemistry (IHC), we observed suppression of miR-320a expression and activation of FoxM1 in 10 cases (71.5%). At the same sites, we observed strong P27^KIP1^ expression, the down-stream target of FoxM1, in the normal tissues and weak expression in the gastric cancer tissues (Figure 1D, 1E). This clinical evidence supports the negative association of miR-320a and FoxM1 expression in gastric cancer. The gene expression was not associated with age, gender, and specimen histology (Supplementary Table S1).

### FoxM1- P27^KIP1^ axis is a direct target of miR-320a

**Figure 2:**
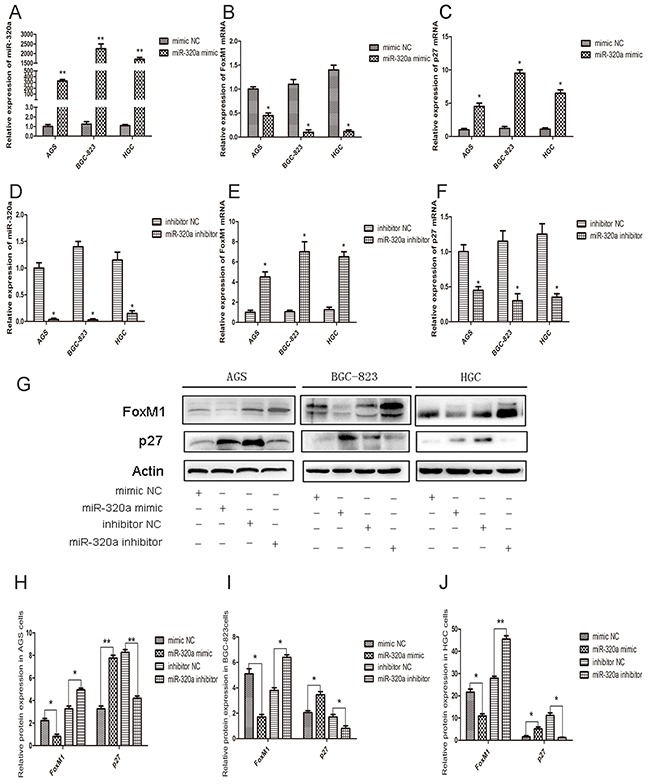
The effect of miR-320a on FoxM1 and P27^KIP1^ expression in human gastric cancer cells qRT-PCR analyses of **A.** miR-320a, **B.** FoxM1 and **C.** P27^KIP1^ mRNA levels in control and miR-320a mimics-transfected AGS, BGC-823 and HGC-27cell lines after 48 h. *P < 0.05 and **P < 0.01. **D.** miR-320a, **E.** FoxM1 and **F.** P27^KIP1^ mRNA levels in control and miR-320a inhibitors-transfected AGS, BGC-823 and HGC-27 cells after 48 h. *P < 0.05 and **P < 0.01. **G.** Western blot analyses of FoxM1 and P27^KIP1^ protein levels in gastric cancer cells treated with control and miR-320a mimics or inhibitors. **H, I, J.** Western blot analyses of FoxM1 and P27^KIP1^ protein levels. *P < 0.05 and **P < 0.01. Data are mean ± SEM of 3 independent experiments. The “mimic NC” means “mimic negative control”, and the “inhibitor NC” as “inhibitor negative control”.

The effect of miR-320a on FoxM1 expression was evaluated using overexpression of miR-320a mimics and inhibitors. In AGS, BGC-823, and HGC-27 cells, the overexpression of miR-320 resulted in the inhibition of FoxM1 mRNA expression and the recovery of P27^KIP1^ expression (Figure 2A, 2B and 2C). Since these cells are at different differentiation stages, they have different transfection efficiency with miR-320a mimics. The protein levels of FoxM1 and P27^KIP1^ were also affected by miR-320a (Figure 2G-2J). On the contrary, the inhibition of miR-320a resulted in the overexpression of FoxM1 and the decreased expression of P27^KIP1^ both at the mRNA and protein levels (Figure 2D-2J).

**Figure 3:**
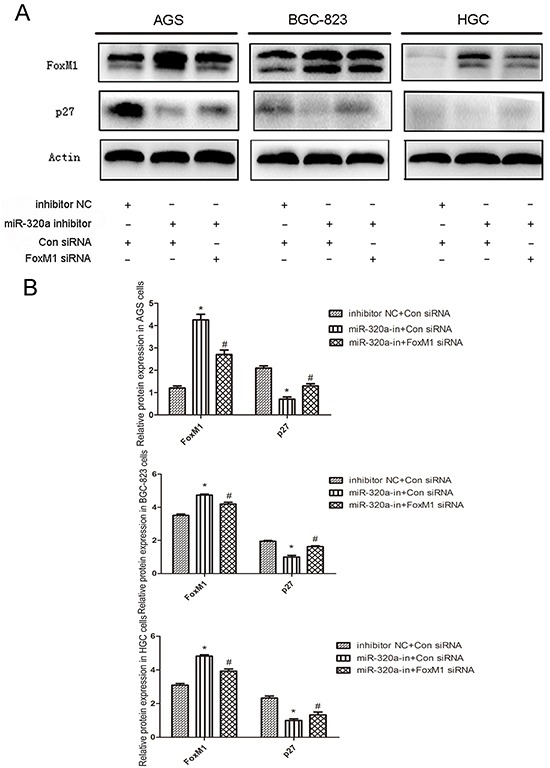
The recovery experiment of miR-320a's regulation of P27^KIP1^ expression through FoxM1in human gastric cancer cells **A.** Western blot analyses of FoxM1 and P27^KIP1^ protein levels in gastric cancer cells treated with control and miR-320a inhibitors or FoxM1 siRNA for 48 h. **B.** Western blot analyses of FoxM1 and P27^KIP1^ protein levels. *P < 0.05 and **P < 0.01. Data are mean ± SEM of 3 independent experiments.

In order to determine whether miR-320a affects the P27^KIP1^ expression by suppressing FoxM1, the recovery experiment was performed. With the transfection of miR-320a inhibitors, the FoxM1 expression increased with P27^KIP1^ inhibition in all the gastric cancer cell lines. While with the co-transfection of miR-320a inhibitors and the special FoxM1 siRNA, both the expression of FoxM1 and P27^KIP1^ recovered to the normal levels (Figure 3A, 3B).

**Figure 4:**
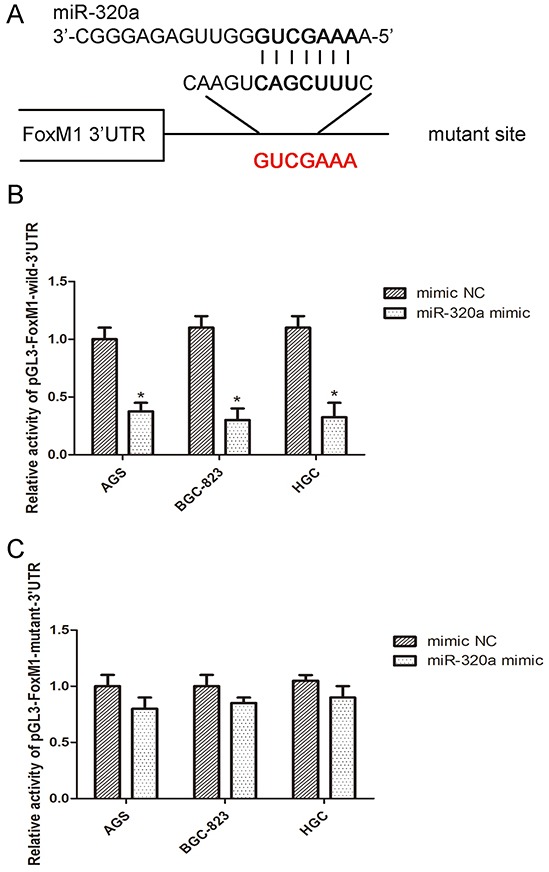
miR-320a directly bound to the 3′-UTR of FoxM1 **A.** The wild sequence on 3′-UTR of FoxM1 that could be bound by miR-320a and the corresponding mutant sequence. **B.** Luciferase activity assay with pMIR-REPORT-FoxM1-3′-UTR co-transfected with miR-320a mimics or the negative control in the three gastric cancer cells for 48h. *P <0.05 vs. con. **C.** Luciferase activity assay with pMIR-REPORT-FoxM1-3′-UTR mutant co-transfected with miR-320a mimics or the negative control in the three gastric cancer cells for 48 h. No significant difference. Data are mean ± SEM of 3 independent experiments.

Next, we validated FoxM1 as a direct target of miR-320a by luciferase report assay. In AGS, BGC-823 and HGC-27 cells, miR-320a mimics were co-transfected with FoxM1 wild-type or mutant-type 3′-UTR plasmids. Co-transfection of miR-320a and wild-type 3′-UTR plasmid reduced the luciferase activity by approximately 62% relative to the control, whereas mutant 3′-UTR co-transfection almost restored the luciferase activity (Figure 4A, 4B and 4C). Thus, miR-320a directly targeted the binding site located at FoxM1 3′-UTR and FoxM1- P27^KIP1^ axis may be a direct target of miR-320a.

### miR-320a regulates proliferation of gastric cancer cells through FoxM1- P27^KIP1^ axis

**Figure 5:**
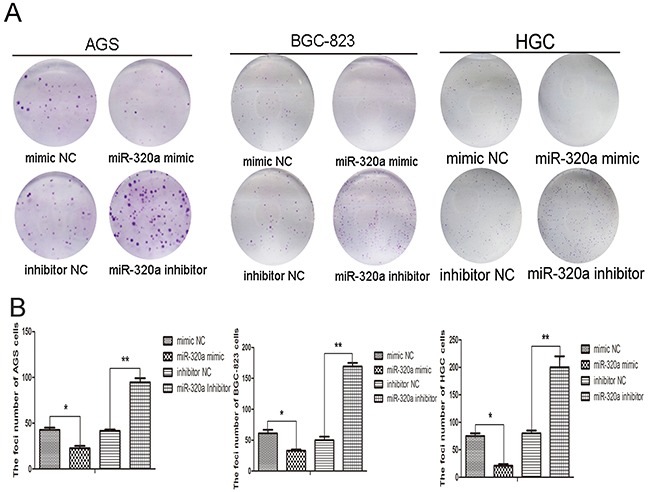
miR-320a was involved in gastric cells proliferation **A.** Colony formation ability in AGS, BGC-823 and HGC-27 cells with overexpression and knockdown of miR-320a and **B.** quantification. *P < 0.05 and **P < 0.01. Data are mean ± SEM of 3 independent experiments.

Colony formation assay in AGS, BGC-823, and HGC-27 cells revealed that enforced expression or knockdown of miR-320a affected cloning of cells (Figure 5A). Transfection of miR-320a mimics reduced the number of colonies, while the inhibition of miR-320a markedly increased the number of colonies (Figure 5B).

**Figure 6:**
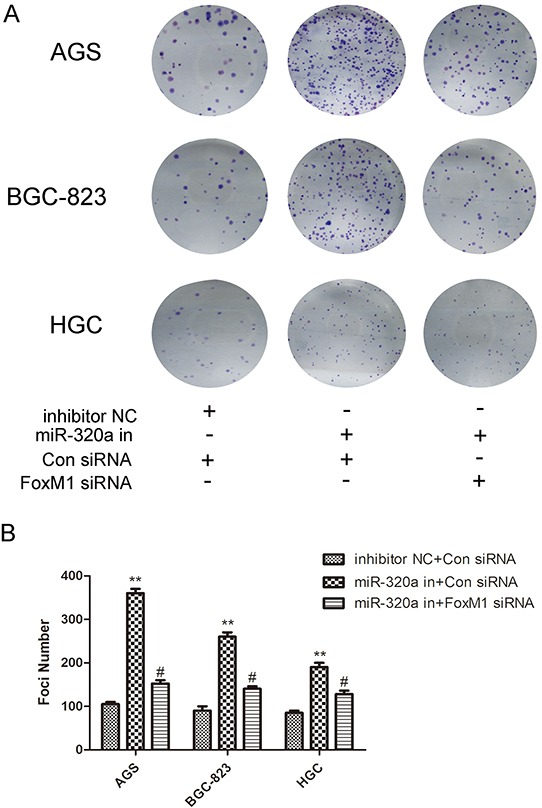
The recovery experiment for miR-320a of colon genetics **A.** Colony formation ability in AGS, BGC-823 and HGC-27 cells with knockdown of miR-320a or co-knockdown of FoxM1 siRNA (5μM) and **B.** quantification. *P < 0.05 and **P < 0.01. Data are mean ± SEM of 3 independent experiments.

The recovery experiment was done also on clone genetics to determine the role of FoxM1- P27^KIP1^ axis in the biological activity of miR-320a. As shown in Figure 6, inhibition of miR-320a resulted in the increase of colonies in all three gastric cancer cell lines, while co-transfection of miR-320a inhibitors and FoxM1 siRNA almost recovered the number (Figure 6A, 6B). Therefore, miR-320a inhibited the proliferation of human gastric cells through the regulation on FoxM1- P27^KIP1^ axis *in vitro*.

### miR-320a suppression increases gastric tumor growth in nude mice through altered FoxM1- P27^KIP1^ signaling

**Figure 7:**
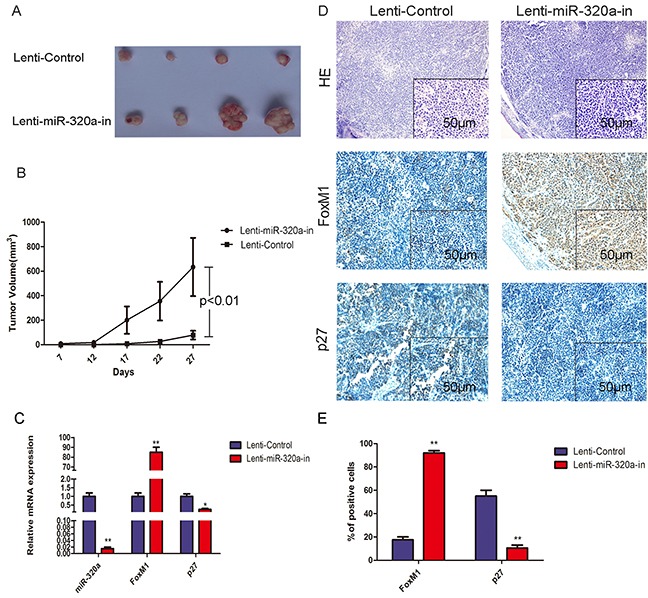
Overexpression of FoxM1 and inhibition of P27^KIP1^ by miR-320a knockdown in nude mice **A.** Tumorigenesis after injection of BGC-823 cells stably expressing miR-320a and controls. **B.** Tumor volume with stably expressing miR-320a and controls. **C.** Growth curve with stably expressing miR-320a and control. **P<0.01. **D.** QRT-PCR analysis of FoxM1 and P27^KIP1^ mRNA expression with stably expressing miR-320a and control. *P<0.05 and **P<0.01. **E.** HE and IHC staining for FoxM1 and P27^KIP1^ in the control group (left panel) and miR-320a inhibitors stable expression group (right panel). Representative images are shown. **F.** Percentage positive area for FoxM1 and P27^KIP1^ expression with the control and miR-320a inhibitors stable expression determined immunohistochemically. **P<0.01.

To evaluate the effect of miR-320a knockdown on the gastric tumor growth *in vivo*, we established stable lentiviral-miR-320a inhibitor-transfected BGC-823 cells and injected them subcutaneously into nude mice. In contrast to control cells, lentiviral-miR-320a inhibitor-transfected BGC-823 cells produced much larger gastric tumors with faster growth (Figure 7A, 7B). To identify the mechanisms involved in miR-320a-associated gastric tumor growth, we examined the effect of miR-320a inhibition on FoxM1 and P27^KIP1^ expression in the tumors. QRT-PCR and IHC analyses demonstrated that silencing of miR-320a increased expression of FoxM1 and decreased expression of P27^KIP1^ (Figure 7C, 7D and 7E). These results suggest that miR-320a suppression increases the tumor growth through altered FoxM1- P27^KIP1^ signaling.

## DISCUSSION

In this study, we demonstrate that miR-320a has an anti-tumor role in gastric cancer. miR-320a directly inhibits the expression of FoxM1 through the binding of FoxM1 3′-UTR, resulting in the increased expression of P27^KIP1^. Our *in vitro* and *in vivo* data indicate that the biological activity of miR-320a is to inhibit the gastric cancer cell proliferation.

Approximately 72% of gastric cancer occurs in developing countries, especially in Asia (e.g. Korea, Japan, and China) and parts of South America [25]. Approximately two thirds of gastric cancer patients show tumor metastasis or local invasion at diagnosis [26]. Surgery is the treatment of choice for early stages of the disease. However, the median survival time is only 6 to 9 months for later stage of disease after surgical treatment [27]. Thus, early detection and treatment of gastric cancer are the key to prolong patients’ survival. Identification and evaluation of novel molecules that are involved in the different stages of gastric cancer genesis and development are important to improve diagnosis and management of gastric cancer.

An extensive amount of research has led to the identification of miRNAs as important regulators of gene expression in cancer biology [28, 29]. Different types of miRNAs can play different roles in gastric carcinoma, such as cell survival, apoptosis, proliferation and cell death [30–33]. Microdissection and microarrays detected different mRNAs and miRNAs in human gastric cancer samples and normal tissues. Some pathways identified in gastric carcinoma, such as Ras-MAPK and Wnt/β-catenin signaling pathways, were also found in this study. We also found an important miRNA, miR-320a, in this study. There are few studies of the function of miR-320a, including its inhibition of ARDP-19/ERRr in breast cancer and targeting ITGB3 in bladder carcinoma [19, 20]. It also functions in atherogenesis and survivin/apoptosis [34, 35]. Bioinformatics analysis indicated that the target of miR-320a is FoxM1. Aberrant expression of FoxM1 is involved in several tumor types, including hepatocellular carcinoma, basal cell carcinoma, breast cancer, lung cancer, prostate cancer, glioblastomas, and gastric cancer, suggesting its oncogenic role in carcinogenesis [36]. Some miRNAs have been identified to regulate the expression of FoxM1, including miR-149, miR-134, miR-370, miR-494, miR-194, and miR-24-1 [37–43]. Thus, we analyzed the expression of miR-320a and FoxM1 in human gastric cancer species. The results of qRT-PCR and IHC showed that the expression of miR-320a was decreased in gastric cancer samples and negatively correlated with FoxM1 expression. Our previous study has shown that P27^KIP1^, one of the important inhibitors of cell cycle and a tumor suppressor, is the down-stream target of FoxM1 and regulated by FoxM1 activity [17]. The results in human samples also showed that miR-320a expression negatively correlated with FoxM1 expression but positively with P27^KIP1^. The luciferase assay and the recovery experiment further revealed that miR-320a controlled the expression of P27^KIP1^ through directly binding to the 3′-UTR of FoxM1, thus inhibiting FoxM1 expression. The biological activity of miR-320a was examined *in vitro* and *in vivo*. The results of clone genetics and the recovery experiment showed that the inhibition of cell proliferation with miR-320 overexpression was through the regulation on FoxM1- P27^KIP1^ axis. The nude mice models confirmed that the inhibition of miR-320a could improve tumorigenesis with overexpression of FoxM1 and decrease of P27^KIP1^. The potential mechanisms of miR-320a's down-regulation of gastric cancer may be diverse. One possibility might be the change of chromosome. Most miRNAs associated with tumors are located in the cancer-associated genomic regions (CAGRs), where they are prone to gene rearrangement, deletion, amplification, etc. [44]. In addition, genetic mutations and single nucleotide polymorphisms (SNP) may also lead to the miRNA's abnormal expression. SNP may affect the transcription of pri-pre-miRNA or produce new binding sites of mRNA-miRNA [45]. Epigenetics could also affect the level of miRNAs. We have previously shown that RBP2, an H3K4 demethylase on di- and tri- methylation, could regulate the expression of miR-21 in gastric cancer [46].

Altogether, our data have revealed a crucial role of miR-320a in limiting the gastric carcinoma by directly targeting FoxM1- P27^KIP1^ axis. Considering the major role of FoxM1 in many types of cancer, miR-320a might be a promising agent to treat other cancers, such as hepatic carcinoma. Based on our findings, we propose that miR-320a might be useful as an anti-gastric cancer therapeutic agent.

## MATERIALS AND METHODS

### Patients and tissue specimens

The study was approved by the Ethics committee of Shandong University School of Medicine (Jinan, PR China). Resected tissues from 22 gastric cancer patients and distal normal gastric tissues (>5 cm from the margin of the tumor) were harvested at surgery. The patients underwent the surgeries at Qilu Hospital, Shandong University (Jinan, PR China) during 2014 and 2015. None of the patients had received adjuvant chemotherapies before surgery. The diagnosis of all gastric cancers was histopathologically confirmed by examination of the surgical specimens. Microdissection (PALM MicroBeam, ZEISS, Germany) and microarray (Exiqon LNATM microRNA and Roche-NimbleGen, KANGCHEN, Shanghai, PR China) were used for sample analysis. Five normal and five gastric cancer samples from Qilu Hospital, Shandong University (Jinan, PR China) were analyzed. Total RNA was harvested using TRIzol (Invitrogen) and RNeasy mini kit (QIAGEN) according to manufacturer's instructions. After having passed RNA measurement on the Nanodrop instrument, the samples were labeled and detected by KANGCHENG using the miRCURY™ Hy3™/Hy5™ Power labeling kit and hybridized on the miRCURY™ LNA Array (v.14.0). The samples were hybridized on a hybridization station. The scanning was performed with the Axon GenePix 4000B microarray scanner. GenePix pro V6.0 was used to read the raw intensity of the images. The miRNA expression profiling was completed on the samples. The profiling identified that miR-320a was inhibited in human gastric cancer tissues *vs.* the normal controls. Detailed patient and disease characteristics are documented in Supplementary Table S1.

### Animal models

Sixteen (8-10 weeks old) male nude mice were purchased from QING ZI LAN Animal Company (Nanjing, China) and divided into two groups with one as control and the other as miR-320a inhibition. The mice were subcutaneously injected with 4×10^5^ BGC-823 cells per mouse. One group was injected with miR-320a inhibitor stable-transduction cells and the other group was injected with the matched control cells. Two weeks later, the mice were sacrificed and the tumor tissues were harvested and photographed. Tissue sections were attained with traditional method and HE staining was performed.

### Cell lines and cell culture

The gastric epithelial-derived cell lines, AGS, BGC-823 and HGC-27 were used for the study. AGS cells were cultured in F12 medium supplemented with 10% fetal bovine serum (FBS, Gibco, USA). BGC-823 and HGC-27 cells were cultured in RPMI 1640 medium supplemented with 10% FBS. All cells were cultured in 5% CO_2_-air at 37°C. All cells were plated for 18-24 h before biological function detections were performed in 6-well plates.

### RNA extraction and qRT-PCR

Total RNA of tissue samples and cultured cells was extracted using TRIzol reagent (Invitrogen, USA). Quantitative real-time PCR (qRT-PCR) assays were carried out to detect mRNA expression using the First Strand cDNA Synthesis Kit (Fermentas, Canada) and SUBR Premix Ex Taq™ (TaKaRa, Japan) according to the manufacturer's instructions. β-actin expression was used as the control. The sequences of the PCR primers were: FoxM1, 5′-TGCAGCTAGGGATGTGAATCTTC-3′ (Forward) and 5′-GGAGCCCAGTCCATCAGAACT-3′ (Reverse); p27, 5′-ATGTCAAACGTGCGAGTGTCTAA-3′ (Forward) and 5′-TTACGTTTGACGTCTTCTGAGG-3′ (Reverse). QRT-PCR analyses for miRNAs were performed by using TaqMan miRNA assays (Applied Biosystems, USA). U6 small nuclear RNA was used as endogenous control for data normalization. Real-time PCR was carried out in an ABI7500 sequence detector (Applied Biosystems, USA). All RT-PCR reactions were conducted in triplicates, and relative quantification was calculated by the 2^−ΔΔCt^ method (95% confidence interval) with calibration to the corresponding endogenous control.

### Western blotting and immunohistochemistry (IHC)

Immunoblotting was performed according to standard western blot procedures. Briefly, proteins (20 mg) were separated by 10% SDS-PAGE and transferred to PVDF membrane (Bio-Rad, USA). After blocking in 5% nonfat milk, the membranes were incubated with the primary antibodies, FoxM1 (1:1000) and P27^KIP1^ (1:300), followed by 1h incubation at RT with the corresponding secondary antibody (1:1000). β-actin was used as a loading control.

FoxM1 expression in tissue specimens was detected by IHC staining according to standard protocols. For incubation with primary antibodies (FoxM1 1:200 and P27^KIP1^ 1:150), tissue slides were incubated at 4°C overnight. Negative controls were treated identically, but without the primary antibody. The primary antibodies for p27 and FoxM1 were purchased from Santa Cruz Biotechnology (USA): p27(C-19), CAT.# sc-528 and FoxM1(H-19), CAT.# sc-501.

### Luciferase assay and transfection

The mimics and the inhibitor of miR-320a were purchased from Ruibo (Guangzhou, PR China). FoxM1 siRNA was purchased from Sigma (USA). The special fragment of the FoxM1 3′-UTR containing the miR-320A predicted target site was synthesized by Invitrogen (USA). Then the fragment was cloned into the multiple cloning sequence of the luciferase reporter pMIR-REPOTR (Applied Biosystems, USA), designated as pMIR-REPORT-FoxM1-3′-UTR (with the binding site CAGCUUU), which was also used in PCR to generate pMIR-REPORT-FoxM1-3′-UTRmut plasmid with mutation of the binding sites on the 3′-UTR of FoxM1(with the binding site mutation GUCGAAA). To examine the direct conjugation of miR-320a to the 3′-UTR of FoxM1, pMIR-REPORT- FoxM1-3′-UTR and pMIR-REPORT- FoxM1-3′-UTRmut were co-transfected into AGS, BGC-823 and HGC-27 cell lines with miR-320a mimics. pMIR-REPORT β-gal plasmid was used as a negative control. Luciferase activity in the cell lysates was determined by a single luciferase reporter assay (Promega, USA) 48 h after transfection, and target promoter-driven firefly luciferase activity was normalized to that of β-gal.

For transient transfection, cells were seeded in 6-well plates (3×10^5^cells/well) for 18 to 24 h, then transfected with plasmids/miRNA mimics and inhibitors/FoxM1 siRNA by the use of Lipofectamine 3000 (Invitrogen, USA) with the standard protocol.

### Clone formation assay

AGS, BGC-823, and HGC-27 cells were incubated in 6-well plates for 18-24 h, and transfected with the corresponding plasmids/ miRNA mimics and inhibitors for 48 h. Single cells were seeded on 6-well plates (300-500 cells/well). After 10-14 days of incubation, plates were stained with Giemsa for 20 min. The number of colonies with more than 50 cells was counted.

### Statistical analysis

FoxM1, P27^KIP1^ and miR-320a expression in different tissue samples analyzed by qRT-PCR and IHC was evaluated by One-Way ANOVA. The comparison of FoxM1, P27^KIP1^ and miR-320a expression, and foci numbers after different treatments was made with a Student's *t*-test. All the tests were determined using the 2-tailed Student *t* test in SPSS software, version 15.0. Results are expressed as the mean ± standard error of the mean. *P* values of <0.05 were considered to be statistically significant.

## SUPPLEMENTARY TABLE


