# Supplementary material for: MiR-320a inhibits gastric carcinoma by targeting activity in the FoxM1-P27KIP1 axis
Source: Oncotarget. 2016 Apr 11;7(20):29275–86. doi: 10.18632/oncotarget.8676 (PMC5045395; doi:10.18632/oncotarget.8676)
Supplement: Supplementary file 1 [file oncotarget-07-29275-s001.pdf]

SUPPLEMENTARY TABLE

Supplementary Table S1: Association of miR-320a and FoxM1 expression in human gastric cancer tissues.

Category	No. of patients	miR-320a			FoxM1		
		high	low	*P*	high	low	*P*
Age				>0.05			>0.05
< 60 years	9	3	6		8	1	
≥60 years	13	5	8		10	3	
Sex				>0.05			>0.05
Male	14	5	9		8	6	
Female	8	3	5		6	2	
Differentiation				>0.05			>0.05
Well	10	4	6		7	3	
Poor	12	4	8		8	4	
Tumor size				<0.05			<0.05
≤5cm	10	8	2		3	7	
>5cm	12	3	9		10	2	
